# The angiogenic potential of CD271+ human adipose tissue-derived mesenchymal stem cells

**DOI:** 10.1186/s13287-021-02177-0

**Published:** 2021-03-02

**Authors:** Richard J. P. Smith, Alessandro Faroni, James R. Barrow, Jamie Soul, Adam J. Reid

**Affiliations:** 1 Blond McIndoe Laboratories, Division of Cell Matrix Biology and Regenerative Medicine, School of Biological Sciences, Faculty of Biology, Medicine and Health, The University of Manchester, Manchester Academic Health Science Centre, Manchester, M13 9PT UK; 2 Biosciences Institute, Faculty of Medical Sciences, Newcastle University, Newcastle Upon Tyne, UK; 3 Department of Plastic Surgery & Burns, Wythenshawe Hospital, Manchester University NHS Foundation Trust, Manchester Academic Health Science Centre, Manchester, UK

**Keywords:** Adipose tissue-derived mesenchymal stem cells (AD-MSCs), Stromal vascular fraction (SVF), CD271, Angiopoietin, Fat grafting, Magnetic-activated cell sorting (MACS), Adipose tissue engineering

## Abstract

**Background:**

Autologous fat grafting is often a crucial aspect of reconstructive and aesthetic surgeries, yet poor graft retention is a major issue with this technique. Enriching fat grafts with adipose tissue-derived mesenchymal stem cells (AD-MSCs) improves graft survival—however, AD-MSCs represent a heterogeneous population. Selection of subpopulations of AD-MSCs would allow the targeting of specific AD-MSCs that may benefit fat graft survival more than the general AD-MSC population.

**Methods:**

Human AD-MSCs were selected for the surface marker CD271 using magnetic-activated cell sorting and compared to the CD271 negative phenotype.  These subpopulations were analysed for gene expression using Real-Time qPCR and RNA sequencing; surface marker characteristics using immunostaining; ability to form tubules when cultured with endothelial cells; and gene and protein expression of key angiogenic mediators when cultured with ex-vivo adipose tissue.

**Results:**

Human AD-MSCs with the surface marker CD271 express angiogenic genes at higher levels, and inflammatory genes at lower levels, than the CD271− AD-MSC population. A greater proportion of CD271+ AD-MSCs also possess the typical complement of stem cell surface markers and are more likely to promote effective neoangiogenesis, compared to CD271− AD-MSCs.

**Conclusion:**

Enriching grafts with the CD271+ AD-MSC subpopulation holds potential for the improvement of reconstructive and aesthetic surgeries involving adipose tissue.

**Supplementary Information:**

The online version contains supplementary material available at 10.1186/s13287-021-02177-0.

## Background

Human adipose tissue-derived mesenchymal stem cells (AD-MSCs) have been investigated as a therapeutic cell population for a number of potential clinical applications including autologous fat transplantation or fat grafting [1]. The addition of autologous cell therapy has been investigated in an effort to improve tissue survival during fat grafting, with mixed success [2]. The stromal vascular fraction (SVF), easily derived from human adipose tissue (AT), contains AD-MSCs, among many other cell types, and exhibits regenerative potential for various indications including burns, nerve injury, and fractures [3]. Underpinning this potential, AD-MSCs have been shown to release several growth factors in vitro, including the angiogenic markers hepatocyte growth factor (HGF), transforming growth factor beta (TGF-β), and vascular endothelial growth factor A (VEGFA), at levels that assist recovery from ischemia in mice and could potentially improve tissue survival [4]. Accordingly, cultured AD-MSCs have been shown to positively influence in vitro endothelial tubule formation via microvesicle signalling [5].

The highly heterogeneous nature of the SVF means this cell population could be refined for particular clinical applications. Endothelial, immune, and blood cells are unlikely to provide support to a de-vascularised adipose tissue graft, and death of these cells in the graft may counter the trophic support of AD-MSCs [6, 7]. As such, we propose that a more targeted therapeutic approach is required, whereby subpopulations of cells from the SVF are selected for their angiogenic characteristics.

Previous studies have highlighted CD271+ AD-MSCs as a subpopulation of interest for therapeutic application. Also known as low-affinity nerve growth factor receptor (LNGFR), or p75, CD271 is a receptor found highly expressed in the glial cells of the central and peripheral nervous systems [8, 9]. CD271 has for many years been a popular target for the isolation of bone marrow-derived mesenchymal stem cells (BM-MSCs), as selection for CD271 effectively eliminates endothelial and haematopoietic cells from the BM-MSC population [10]. AD-MSCs expressing CD271 have been found to also express Notch1, Rex1, and Nestin, markers of self-renewal, pluripotency, and cytoskeletal competency respectively [11–13]. In human AT, the expression of CD271 is variable between patients, but typically between 4 and 20% of the extracted AD-MSC population expresses CD271, as measured by flow cytometry [14–16]. Although CD271 expression in AD-MSCs in vivo has been shown to decrease with age, the expression is still higher than that found in BM-MSCs [17].

Here, we show that CD271+ AD-MSCs hold great potential in tissue engineering applications. CD271+ AD-MSCs exhibit increased expression of angiogenic markers, are more likely to belong to the typical MSC phenotype CD45−/CD90+, produce more complex vascular networks, and contribute to a more angiogenic environment when grown with AT, compared to CD271− AD-MSCs.

## Materials and methods

### Adipose tissue sourcing and harvest

Abdominal adipose tissue was harvested from donor patients undergoing DIEP flap breast reconstruction at Wythenshawe Hospital, Manchester University NHS Foundation Trust, UK, after informed consent was granted. Ethics approval was given from the NHS Health Research Authority, National Research Ethics Service Committee South Central – Hampshire B: reference 13/SC/0499. Fresh adipose tissue was minced into a homogenous pulp using surgical scissors and razor blades. The pulp was divided into 50-ml falcon tubes with an equal volume of 0.2% (w/v) collagenase I in HBSS and agitated in a 37 °C water bath for 1 h. The resulting digested tissue was filtered through a 100-μm nylon mesh (Merck Millipore UK Ltd., Watford, UK), and to this was added an equal volume of α-Minimum Essential Medium Eagle (αMEM, Sigma Aldrich, Poole, UK, always containing unless otherwise stated: 10% Foetal Bovine Serum (FBS, LabTech, Uckfield, UK), 1% L-Glutamine (GE Healthcare UK Ltd., Little Chalfont, UK), 1% PenStrep). The tubes were centrifuged at 160*g* for 10 min. The pellet (the stromal vascular fraction, SVF) was resuspended in 1 ml Red Blood Cell Lysis Buffer (Sigma Aldrich, Poole, UK) for 1 min; then, 20 ml αMEM was added to arrest lysis. The mixture was centrifuged at 160 g for 10 min, and the resulting pellet was resuspended in freezing mix (FBS + 10% DMSO (dimethyl sulfoxide, Sigma Aldrich, Poole, UK)) and slow-frozen to − 80 °C until further use.

### Magnetic-activated cell sorting (MACS)

Frozen SVF was thawed in a 37 °C water bath and resuspended in 10 ml αMEM. The suspension was passed through a 40-μm cell strainer which was washed with an additional 10 ml αMEM. The cells were counted (Scepter 2.0 automated cell counter, Merck Millipore UK Ltd., Watford, UK) and around 1% of total cells were set aside as the unsorted population. The remaining suspension was centrifuged at 300*g* for 10 min, and the cell pellet resuspended in 60 μl MACS buffer (0.5% Bovine Serum Albumin (BSA, Sigma Aldrich, Poole, UK) and 2 mM Ethylenediaminetetraacetic Acid (EDTA, Sigma Aldrich, Poole, UK) in Phosphate Buffer Saline solution (PBS), 20 μl CD271 microbead solution (Miltenyi Biotec, Surrey, UK, 130-099-023), and 20 μl FcR blocking reagent (Miltenyi Biotec, Surrey, UK, 130-099-023) per 10^7^ cells and incubated for 15 min at 4 °C. The cells were then washed with 1 ml MACS buffer per 10^7^ cells and centrifuged at 300*g* for 10 min and resuspended in MACS buffer. The magnetic column (Miltenyi Biotec, Surrey, UK, 130-042-401) was attached to a MACS magnet (Miltenyi Biotec, Surrey, UK, 130-090-976), and the cell suspension was passed through the column, with flow-through collected as the negative population (CD271− AD-MSCs). Subsequently, the column was removed from the magnet, and the remaining cells flushed out of the column using the plunger: this was the positive population (CD271+ AD-MSCs). At least 200,000 cells for each group were used for flow cytometry analysis.

### Flow cytometry

Cells from the sorting procedure were centrifuged at 300*g* for 10 min and pellets resuspended in the appropriate fluorescent antibodies or isotype control antibodies at a concentration of 1:11 in MACS buffer. Cells were incubated in up to three antibodies for 10 min at 4 °C. After a final wash step, cells were resuspended in MACS buffer and transported directly for flow cytometry and analysed for surface marker expression using a Cyan ADP flow cytometer (Beckman Coulter, High Wycombe, UK) at the Faculty of Biology, Medicine and Health Core Facility, University of Manchester. Compensation was carried out using a bead kit (Miltenyi Biotec, Surrey, UK, 130-097-900). All antibodies and isotype controls used were obtained from Miltenyi Biotech (Surrey, UK) and are as follows with product codes: CD29-PE (130-101-275), CD34-PE-Vio770 (130-100-844), CD45-PerCP (130-098-145), CD90-FITC (130-097-930), CD146-VioBlue (130-099-678), CD271-APC (130-091-884), Mouse IgG1-PE (130-098-845), Mouse IgG2a-PE-Vio770 (130-098-564), Mouse IgG2a-PerCP (130-099-190), Mouse IgG1-FITC (130-098-847), Mouse IgG1-VioBlue (130-099-756), and Mouse IgG1-APC (130-098-846). Data were analysed using FlowJo v10 (FlowJo LLC, Ashland, OR, USA).

### Human AD-MSC culture

Both sorted and unsorted AD-MSCs were cultured in the same manner, in T75 flasks at 37 °C, 5% CO_2_, with three media changes of 10-ml fresh αMEM every week. The protocol for passaging cells was as follows: HBSS wash, 5 min in 3 ml 0.25% Trypsin (Life Technologies Ltd., Paisley, UK), addition of 5 ml αMEM, centrifugation at 900 rpm for 5 min, resuspension of pellet in αMEM, and replating. Observation of cultured AD-MSCs was achieved using a light microscope (Olympus IX51).

### Immunocytochemistry

Staining for CD271 and CD31 was carried out on tissue fixed with paraformaldehyde (PFA). Immediately upon receipt of human tissue, small 0.5-cm^3^ chunks of tissue were incubated in 4% PFA for 24 h, before being washed three times with PBS-Sucrose for 24 h/wash. Tissue was then embedded in OCT matrix (Fischer Scientific, Loughborough, UK) and flash-frozen with liquid nitrogen. Tissue was cryosectioned onto glass slides in 30-μm slices and dried at 37 °C overnight. Slides were rehydrated in PBS for 10 min before being washed in 0.2% Triton X-100 in PBS for 1 h. Slides were blocked with the appropriate serum (Normal Goat Serum 1:100 (Sigma Aldrich, Poole, UK) in antibody diluent (1% BSA, 1% Sodium Azide, 0.3% Triton X-100 in PBS)) for 1 h at room temperature, washed with PBS, then incubated in primary antibodies overnight: rabbit monoclonal anti-CD271 antibody (Abcam, UK, ab52987) and mouse monoclonal anti-CD31 antibody (Abcam, UK, ab24590) diluted 1:100 in antibody diluent. Next, slides were washed in PBS and secondary antibody was added (Goat anti-rabbit 488, Thermo Fisher Scientific, UK, A27034, and Donkey anti-mouse 568, Thermo Fisher Scientific, UK, A10037) at 1:500 in antibody diluent. After 2-h incubation at room temperature, and a PBS wash, coverslips were fixed with DAPI-containing Vectashield (Vector Labs, UK, H-1200) to stain for cell nuclei. Slides were viewed under a fluorescent microscope (Olympus BX60) and images recorded in Image Pro Plus (v9.1, Media Cybernetics).

### Real-time qPCR and RNA sequencing

RNA was extracted from sorted and differentiated AD-MSCs using a Qiagen RNeasy plus mini kit (Qiagen, Manchester, UK), and RNA was extracted from frozen adipocytes using a Qiagen RNeasy lipid tissue mini kit (Qiagen, Manchester, UK). cDNA was transcribed from RNA using Qiagen RT2 reverse transcription kit (Qiagen, Manchester, UK). Between 1 and 3 ng cDNA was used in each reaction, kept consistent within each experimental run. The PCR mix within each individual reaction consisted of 1 μl of sample, along with 12.5 μl RT^2^ SYBR green mastermix (Qiagen, Manchester, UK), 1 μl primer mix (primers shown in Supplemental Table 1) and made up to 25 μl total with RNase-free water. Samples were run in a Corbett Rotor-Gene 6000 with the following protocol: 10 min at 95 °C, 45 cycles of 15 s at 95 °C, 30 s at 60 °C and 30 s at 72 °C, then hold at 95 °C for 1 min, 65 °C for 2 min, then melting curve from 65 to 95 °C at 2 °C/min. Analysis was performed in the Rotor-Gene’s analysis software using the ∆∆C_T_ method. Relative expression in individual samples was normalised to 18 s, and changes between samples were normalised to either CD271− AD-MSCs (characteristics analysis, see the ‘Results’ section, ‘Identification, extraction, and characterisation of CD271+ AD-MSCs’) or CD271− AD-MSCs with AT (co-culture analysis, see the ‘Results’ section, ‘Co-culture of CD271+ AD-MSCs with AT’).

RNA sequencing was carried out with the assistance of the Bioinformatics core facility at the University of Manchester. Samples were sorted using MACS as above, then preserved in RNA Later (Qiagen, 76106), and immediately sent to the core facility for RNA extraction and analysis. Briefly, reads were trimmed with Trimmomatic v0.36, mapped to the reference genome using STAR v2.5.1a, ensEMBL v90, and Gencode v27 (freeze date 01/27, GRCh38). Gene count was achieved using HTSeq-count v0.6.1p1 and normalised using DESeq2 v1.10.0, allowing samples to be directly compared.

### Reactome pathway analysis

Differentially expressed genes (1.5 absolute fold change and Benjamini–Hochberg-corrected *p* values ≤ 0.05) were used with the R package GOSeq to identify differentially regulated pathways. Pathways with Benjamini–Hochberg-corrected *p* values of ≤ 0.05 were regarded as statistically significant [18, 19].

### HUVEC tubule formation assay

Human umbilical vascular endothelial cells (HUVEC) were acquired from Merck Millipore (SCCE001) and plated according to instructions at 5000 cells per cm^2^ in T75 flasks. Cells were grown in standard culture conditions (see above). Media used was EndoGro-LS from Merck Millipore (SCME001). Upon reaching confluence, HUVEC cells were trypsinised and plated on 24-well plates coated with Matrigel, at a seeding density of 16,000 cells per well. Matrigel coating was achieved using a 1:1 dilution of Matrigel (Corning UK, 356234) in EndoGro-LS media. Once HUVEC cells were plated in the Matrigel-coated wells, AD-MSCs were sorted and seeded with the HUVEC cells at a density of 8000 cells per well. Cells were cultured in EndoGro-LS media for 16 h, before images were recorded using light microscopy. The average tubule length was recorded using the Image J (v1.47f) ‘measure’ tool. Average tubule lengths were calculated, with at least ten images from each experimental group. Number of anastomoses was counted.

For co-staining of HUVECs and AD-MSCs, the above procedure for growing the cells together was followed, although with pre-labelled red fluorescent protein (RFP) expressing HUVEC obtained from Angio-Proteomie (cAP-0001RFP) at passage 3, and passage 3 CD271+ AD-MSCs stained with carboxyfluorescein succinimidyl ester (CFSE). Images were recorded using fluorescence microscopy.

### Ex vivo co-culture

Following the protocol of Anayama et al., an ex vivo model of AD-MSC/adipose tissue co-culture was set up using special well inserts [20]. Immediately following rough tissue homogenisation with a razor blade, 0.1 ml units of undigested adipose tissue were added to the centre of specialised well inserts (Merck Millipore, PICM03050) sitting in six-well plates. Two millilitres of media (1:1 F12:DMEM mix, 10% FBS, 1% Pen-strep) was added to each well, so that media reached the membrane of the inserts, but did not cover them. Remaining tissue was sorted using the standard MACS protocol. Sorted AD-MSCs were seeded in separate six-well plates at 150,000 cells/well, and grown in the same F12:DMEM mix as the adipose tissue. After 48 h, the inserts containing adipose tissue were transferred across to the wells containing AD-MSCs—this marked day 0 of the co-culture. Media was changed every 2 days, and cells cultured in normal conditions, with the L-glutamate-free F12:DMEM media mix, for 15 days. At the endpoint, AD-MSCs were collected in RNA Later (Qiagen, 76106) for PCR analysis, and media was removed for ELISA.

### ELISA

ELISA kits were purchased from RayBiotech for the analysis of levels of VEGF-A (product ELH-VEGF), HGF (product ELH-HGF), and angiopoietin-1 (product ELH-ANG1) in conditioned media. After 15 days of co-culture, media was collected after 48 h of incubation. A standard curve of target protein was created for each kit, and media was added to the microplates in triplicate and incubated according to the kit’s instructions. Absorbance was read at 450 nm. To calculate protein concentrations, the standard curve was plotted in Graph Pad Prism (v6) and absorbances of unknown samples were interpolated against this curve to produce protein concentration values.

### Statistical analysis

All statistical analysis was undertaken in Graph Pad Prism (v6). One-way or two-way ANOVAs with either Tukey’s or Sidak’s multiple comparisons tests were performed in all cases with more than two comparisons, and unpaired *t* tests were carried out in other cases. All data are presented as means ± SEM. In all cases, significance was assumed when *p* < 0.05, and *p < 0.05, **p < 0.01, ***p < 0.001, ****p < 0.0001.

## Results

### Identification, extraction, and characterisation of CD271+ AD-MSCs

**Fig. 1:**
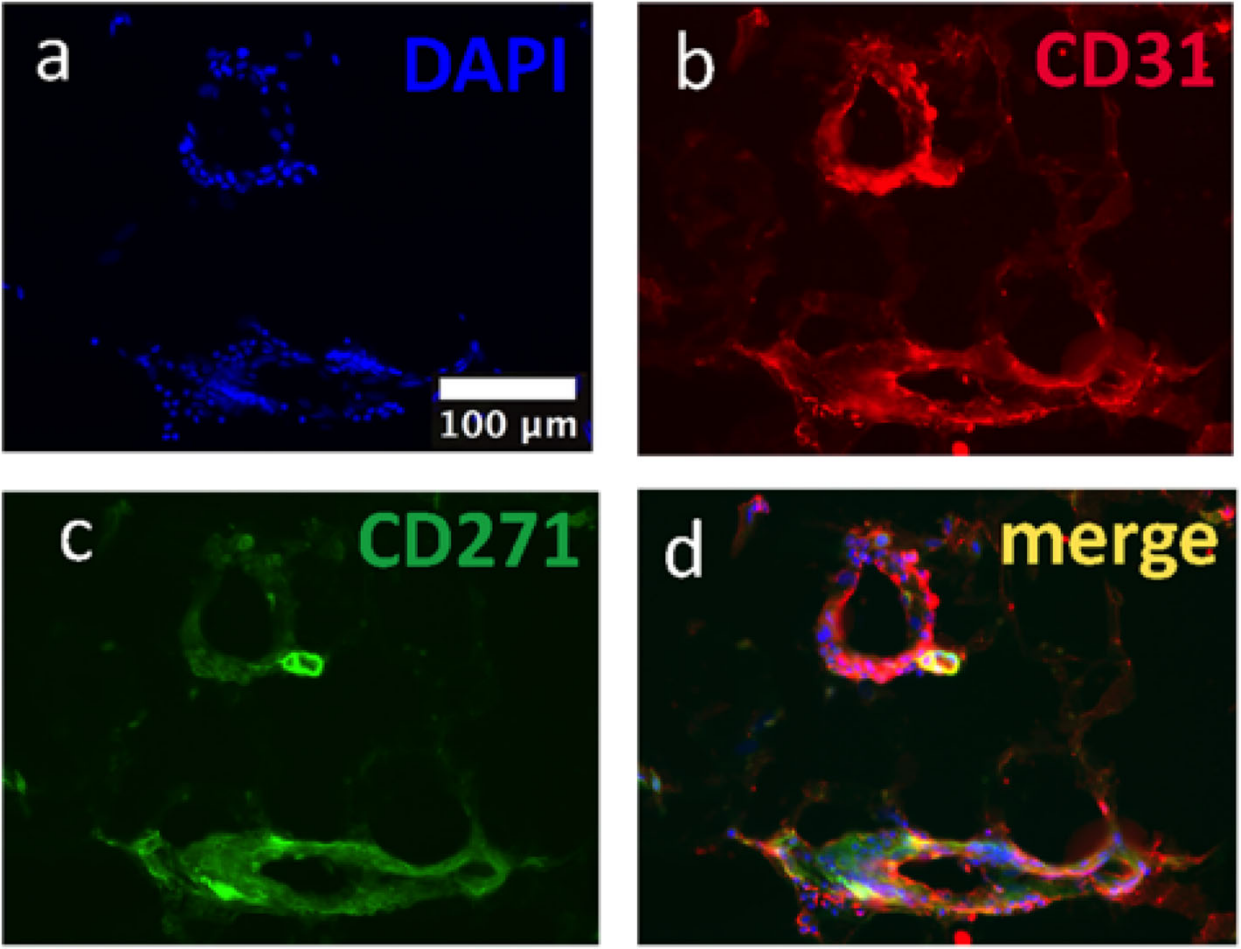
Location of CD271+ AD-MSCs in vivo. Co-staining of fixed human adipose tissue for cell nuclei (**a**), CD31 (**b**), and CD271 (**c**) revealed co-localisation of CD271+ cells with CD31+ cells (**d**), indicating that CD271 cells are mostly localised around vascular structures

Staining of sections of human adipose tissue (AT) revealed that CD271 was found exclusively around vascular structures, confirmed by co-localisation with CD31 staining (Fig. 1), suggesting that CD271+ AD-MSCs are involved in vascular function in vivo.

**Fig. 2:**
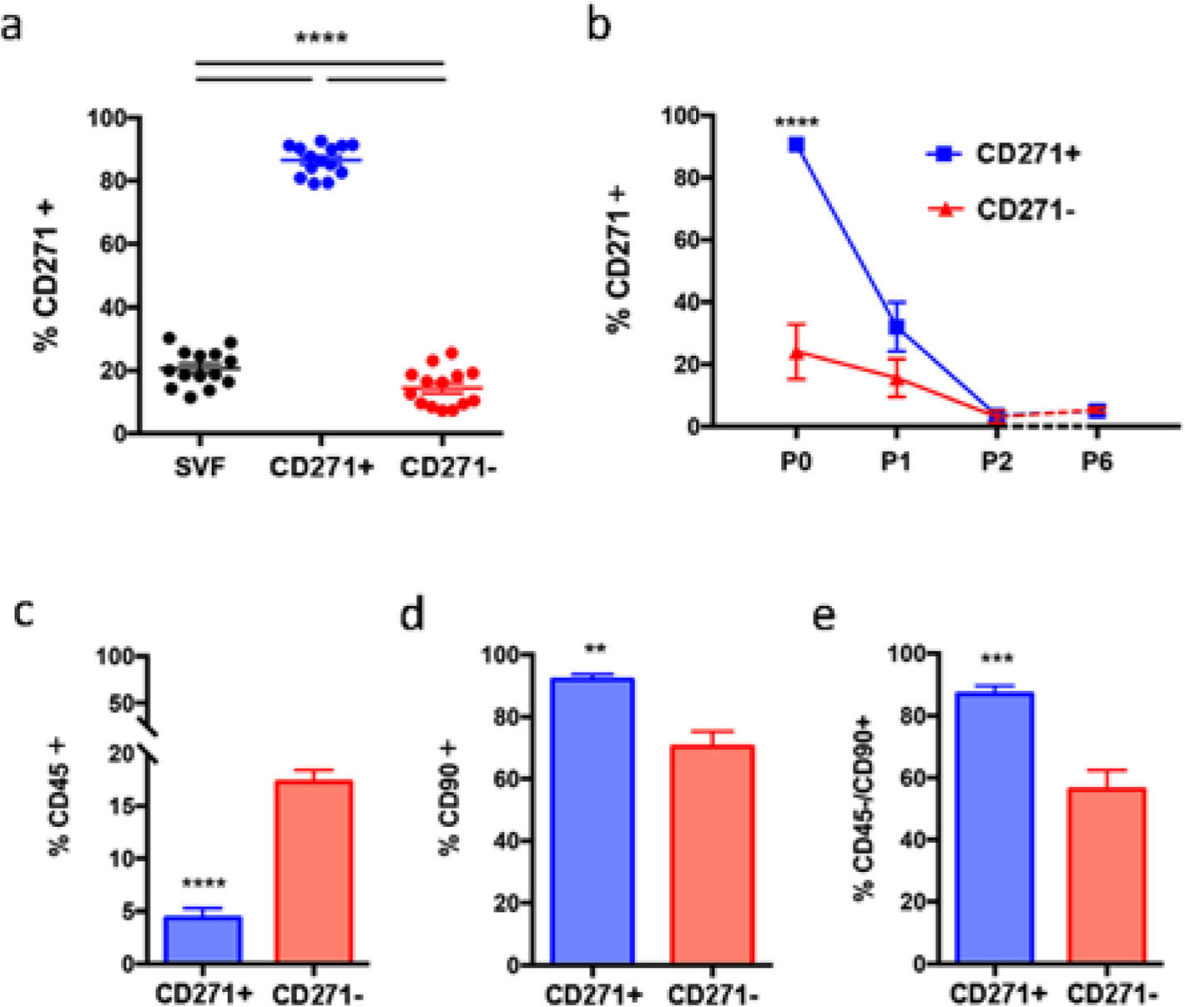
Surface marker expression of AD-MSCs sorted for CD271. AD-MSCs sorted for CD271 were analysed for expression of surface markers using flow cytometry immediately following sorting, and during several passages of cell culture. Expression of CD271 was consistently high in the CD271 purified population (CD271+), compared to the negative sort (CD271−) and the unsorted SVF (**a**, *n* = 14). During cell culture, CD271 expression rapidly decreased in both CD271+ and CD271− populations (**b**, *n* = 4). CD45 expression was significantly lower in CD271+ AD-MSCs (**c**, *n* = 8), and CD90 expression significantly higher (**d**, *n* = 8). The number of CD45−/CD90+ cells was also higher in CD271+ AD-MSCs (**e**, *n* = 8)

Sorting of human SVF using MACS successfully purified a CD271+ population as measured by flow analysis (Fig. 2a). Patients were consistent in the initial proportion of CD271+ AD-MSCs within SVF (average 20.6 ± 1.5%, *n* = 14), and sorting reliably produced a CD271+ population of average 86.6% purity (± 1.3%, *n* = 14). The sorted CD271− population contained an average of 14.5% CD271+ cells (± 1.6%, *n* = 14). Two-way ANOVA analysis with Tukey’s multiple comparisons test revealed that all three groups were significantly different at the *p* < 0.0001 level.

CD271 expression, as measured by flow cytometry, decreased in both sorted populations following cell culture (Fig. 2b). At passage 1, expression in CD271+ cells had dropped to just 25.9% (± 8.3%), which was not significantly different from CD271 expression in passage 1 CD271− cells (12.9 ± 5.1%). Expression continued to drop during subsequent culture, reaching a baseline of around 5% expression in both populations at passage 6 (*n* = 4).

In passage 0 cells, immediately following sorting, typical stem cell markers were higher in CD271+ AD-MSCs (Fig. 2c–e). CD45 was present in low levels in both groups (Fig. 2c), but was significantly lower in CD271+ cells compared to CD271− cells (4.4 ± 0.9% vs. 17.3 ± 1.1%, *p* < 0.0001, *n* = 8). CD90 was present in significantly higher levels in CD271+ cells compared to CD271− cells (Fig. 2d, 91.9 ± 1.8% vs. 70.4 ± 4.9%, *p* = 0.0011, *n* = 8). CD271+ cells contained a significantly higher proportion of CD45−/CD90+ cells compared to CD271− cells (Fig. 2e, 86.8 ± 2.7% vs. 56.2 ± 6.2%, *p* = 0.0005, *n* = 8).

**Fig. 3:**
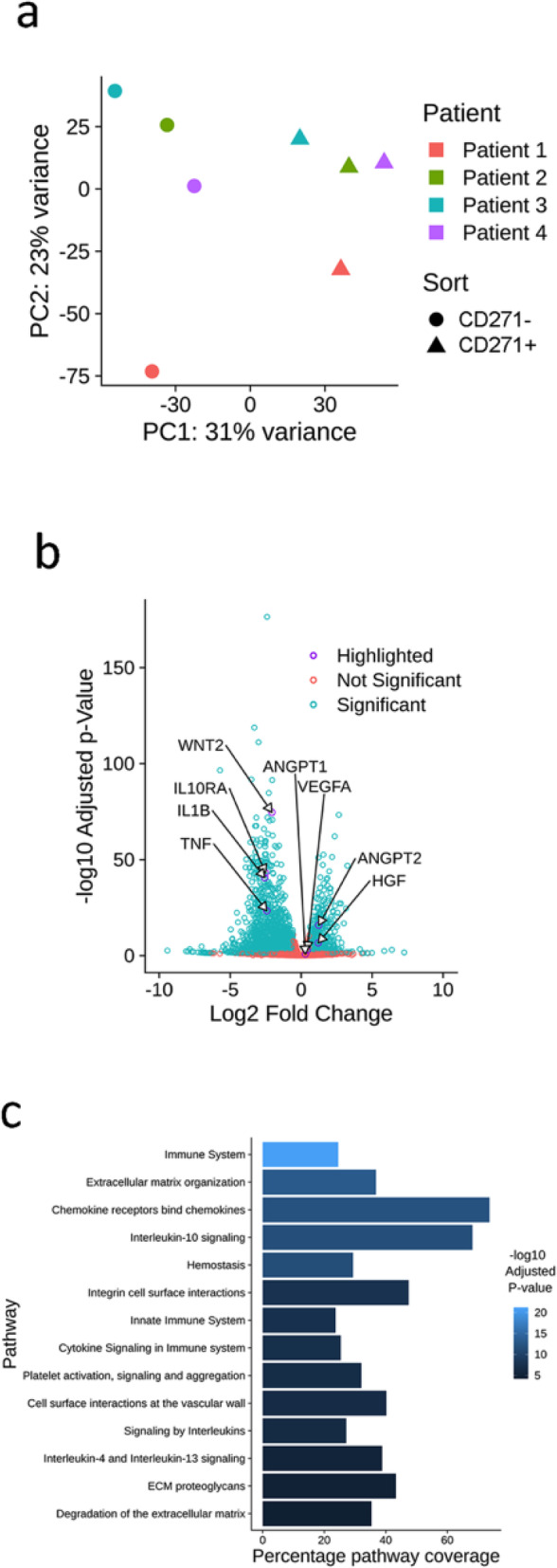
RNA sequencing of CD271+ AD-MSCs. Principal component analysis (**a**) shows the degrees of variance between patients and between CD271+ and CD271− AD-MSCs. The volcano plot (**b**) shows all genes that changed by more than 1.5 fold in the CD271+ AD-MSCs compared to CD271− AD-MSCs. Significance is indicated in blue (significant, *p* < 0.05) and red (non-significant, *p* > 0.05). Data is represented as fold change in expression on the *x*-axis (in log2, with increases on the right hand side of the central ‘0,’ and decreases on the left hand side of the central ‘0.’), against significance as a *p* value on the *y*-axis (in –log10). Reactome analysis (**c**) indicates pathways of interest that are significantly altered in the CD271+ AD-MSCs compared to CD271− AD-MSCs

RNA sequencing revealed significant changes in gene expression between CD271+ and CD271− AD-MSCs (Fig. 3). Principal component analysis confirmed that variation between sorted groups was greater than variation between patients (31% vs. 23% variation, Fig. 3a). Genes of interest are highlighted in Fig. 3b, which displays all genes expressed with greater than 1.5-fold difference in the CD271+ population compared to the CD271− population.

The eight selected genes of interest are also displayed in Table 1 with their respective fold changes and degrees of statistical significance. Angiogenic genes ANGPT2 and HGF were more than doubled in expression in CD271+ AD-MSCs compared to the CD271− population, while angiogenic genes VEGFA and ANGPT1 showed a less significant trend towards higher expression. Inflammatory genes IL10RA, IL1B, and TNF were all reduced in CD271+ cells compared to CD271− AD-MSCs. WNT2, implicated in oncogenesis, was expressed lower in CD271+ cells.

**Table 1:** Expression changes in genes of interest in CD271+ AD-MSCs. Expression changes are presented as fold change in the CD271+ population compared to the CD271− population, by RNA sequencing analysis

Gene	Fold change	Adjusted *p* value	Gene function
ANGPT1	1.22	0.156	Vascular development and angiogenesis
ANGPT2	2.36	1.42E−16	Vascular remodelling, angiogenesis in combination with VEGF
VEGFA	1.34	0.00120	Vascular growth factor
HGF	2.31	2.27E−07	Angiogenic growth factor
IL10RA	0.18	7.55E−44	Inflammatory receptor
IL1B	0.17	2.36E−41	Key cytokine in adipose tissue
TNF	0.19	4.23E−24	Activates M1 macrophages in adipose tissue
WNT2	0.24	2.39E−75	Oncogenic gene

*ANGPT1* angiopoietin 1, *ANGPT2* angiopoietin 2, *VEGFA* vascular endothelial growth factor A, *HGF* hepatocyte growth factor, *IL10RA* interleukin 10 receptor A, *IL1B* interleukin 1B, *TNF* tumour necrosis factor, *WNT2* Wingless-type MMTV integration site family, member 2

Reactome analysis indicated that gene pathways associated with the immune system, extracellular matrix organisation, chemokine receptors, and IL10 signalling were most different between the CD271+ and CD271− AD-MSC populations (Fig. 3c).

Supplemental tables show genes with the highest fold increases and decreases between CD271+ and CD271− AD-MSCs (Supplemental Tables 2 & 3). Notably, expression of NGFR (CD271) is significantly increased by over fourfold in CD271+ cells (*p* = 4.3E−51), as another confirmation of the effectiveness of the technique. Supplemental Table 4 shows more detailed reactome pathway data.

**Fig. 4:**
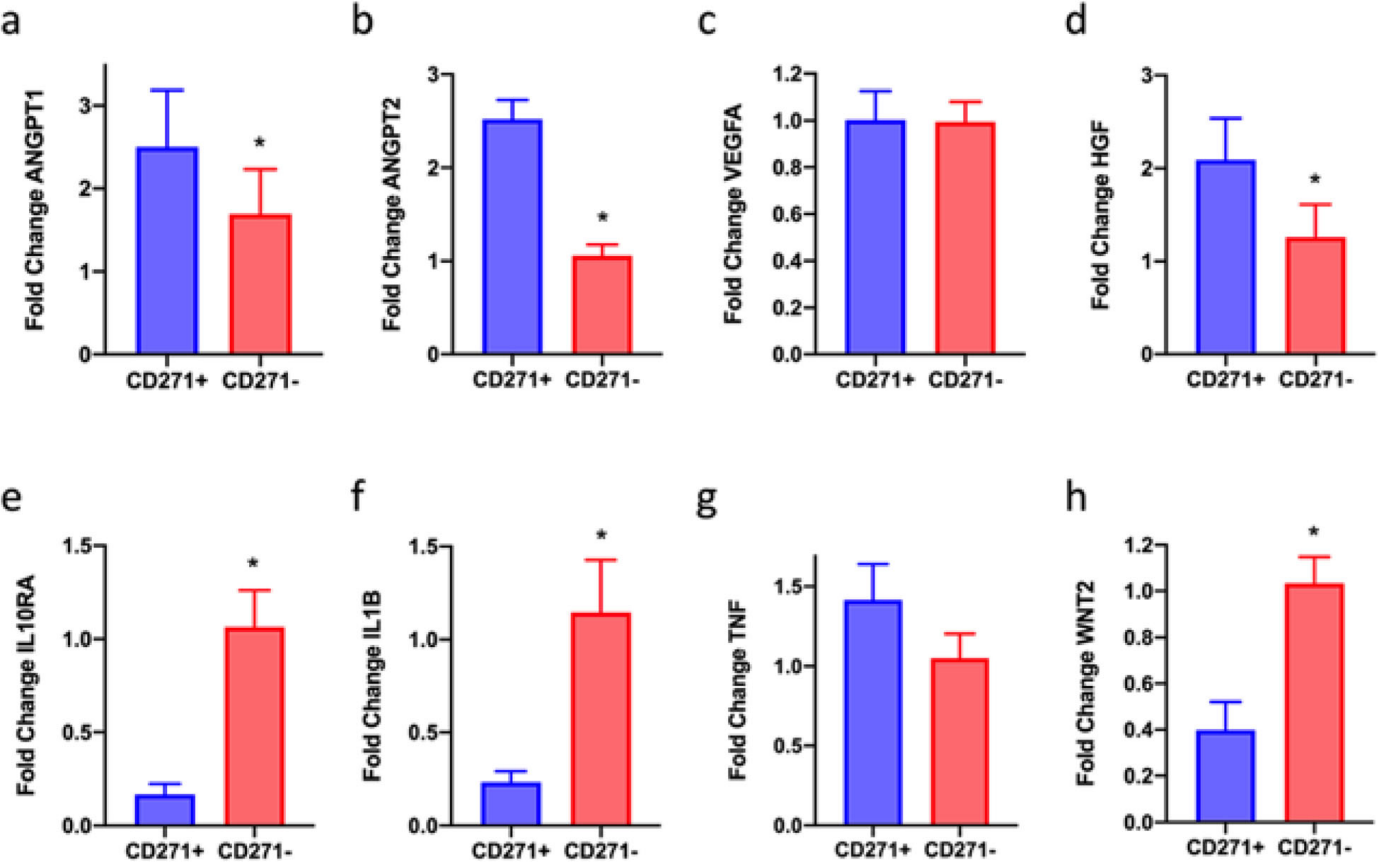
Real-time qPCR analysis of CD271+ AD-MSCs. Analysis of RNA expression in AD-MSCs sorted for CD271, for angiogenic genes (**a**–**d**) and inflammatory/oncogenic genes (**e**–**h**). ANGPT1, angiopoietin 1; ANGPT2, angiopoietin 2; VEGFA, vascular endothelial growth factor A; HGF, hepatocyte growth factor; IL10RA, interleukin 10 receptor A; IL1B, interleukin 1B; TNF, tumour necrosis factor; WNT2, Wingless-type MMTV integration site family, member 2. The ΔΔCT method was used for analysis: all data is normalised to 18 s, and to the between-patients average of CD271− AD-MSCs. *n* = 4

Findings from RNA sequencing analyses were validated using real-time qPCR, which confirmed changes in genes of interest between CD271+ and CD271− AD-MSCs (Fig. 4). Although there was no significant difference in VEGFA expression (Fig. 4c, 1.00 ± 0.12 fold in CD271+ cells, *p* = 0.962, *n* = 4), HGF was expressed higher in CD271+ cells compared to CD271− cells (Fig. 4d, 2.09 ± 0.45 fold, *p* < 0.05, *n* = 4), and both ANGPT1 (Fig. 4a, 2.50 ± 0.68 fold, *p* < 0.05, *n* = 4) and ANGPT2 (Fig. 4b, 2.52 ± 0.21 fold, *p* < 0.05, *n* = 4) were expressed at higher levels, mirroring RNA sequencing results. Expression of both IL10RA and IL1B was lower in CD271+ cells compared to CD271− cells (Fig. 4e, 0.17 ± 0.06 fold, *p* < 0.05, *n* = 4; Fig. 4f, 0.23 ± 0.06 fold, *p* < 0.05, *n* = 4), and WNT2 levels were also decreased in CD271+ cells (Fig. 4h, 0.40 ± 0.12 fold, *p* < 0.05, *n* = 4), again mirroring the RNA sequencing results. Levels of TNF were not significantly changed in this analysis (Fig. 4g, 1.42 ± 0.23 fold, *p* = 0.066, *n* = 4).

### HUVEC tubule formation assay with CD271+ AD-MSCs

**Fig. 5:**
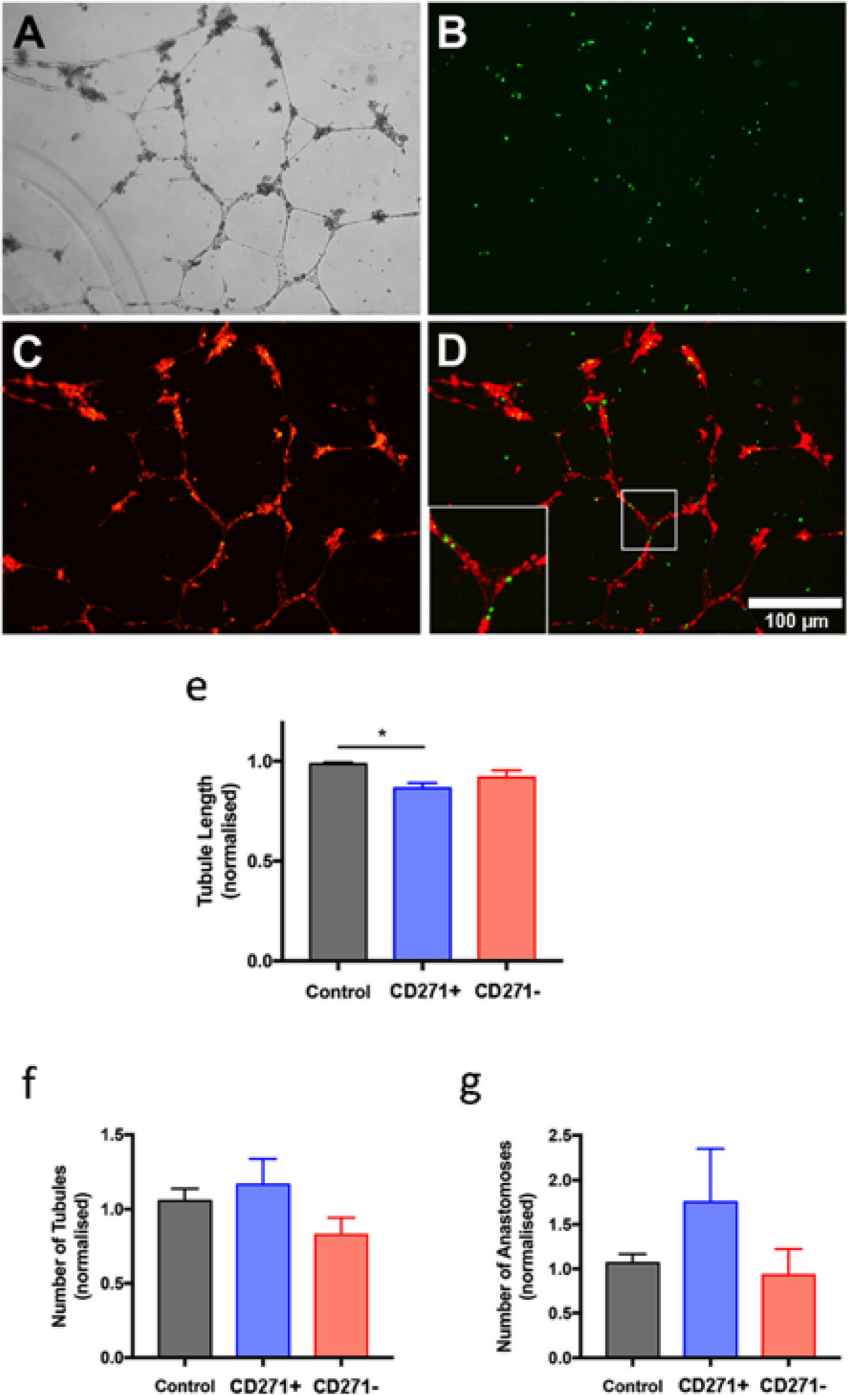
Tubule formation analysis of HUVECs grown with CD271+ AD-MSCs. CD271+ AD-MSCs were grown with HUVECs for 16 h. Light microscopy reveals tubule formation (**a**). Staining the CD271+ AD-MSCs with CFSE (**b**) co-cultured with RFP-HUVECs (**c**) revealed co-localisation along the tubules (**d**, inset). Average tubule length, average number of tubules, and average number of anastomoses were measured after growing HUVECs with either CD271+ AD-MSCs or CD271− AD-MSCs, and data was normalised to HUVEC-only control (**e**–**g**). *n* = 6

RFP-labelled HUVECs and CFSE-stained CD271+ AD-MSCs were grown together for 16 h, and fluorescence microscopy images were recorded to observe the potential involvement of CD271+ AD-MSCs within vascular networks (Fig. 5a–d). Merged imaging shows that CD271+ cells closely associated with tubules (Fig. 5d).

Unstained HUVECs were then grown with unstained CD271+ and CD271− AD-MSCs for 16 h, and characteristics of the resultant tubule network were measured using light microscopy (Fig. 5e–g). Analysis of average tubule length showed that tubule length was slightly shorter when HUVECs were grown with CD271+ AD-MSCs, compared to CD271− AD-MSCs (0.86 ± 0.03 vs. 0.92 ± 0.04, normalised to HUVEC-only control, *p* = 0.336, *n* = 6, Fig. 5e). HUVECs grown with CD271+ AD-MSCs were also significantly shorter than HUVECs grown alone (0.86 ± 0.03 vs. 0.99 ± 0.01, normalised to HUVEC-only control, *p* = 0.013, n = 6, Fig. 5e). There were no significant differences in the number of tubules per field of view between groups, although HUVECs grown with CD271+ AD-MSCs tended to produce more tubules than when grown with CD271− AD-MSCs (1.17 ± 0.17 vs. 0.83 ± 0.11, normalised to HUVEC-only control, *p* = 0.185, n = 6, Fig. 5f). Although there was no significant difference in the average number of anastomoses per field of view between groups, HUVECs grown with CD271+ AD-MSCs had nearly double the number of anastomoses compared to those grown with CD271− AD-MSCs (1.75 ± 0.60 vs. 0.93 ± 0.29, normalised to HUVEC-only control, *p* = 0.321, *n* = 6, Fig. 5g).

### Co-culture of CD271+ AD-MSCs with AT

Following 15 days of indirect co-culture between AD-MSCs and ex-vivo AT, levels of angiogenic protein in the media (ELISA) and expression of angiogenic genes in the AD-MSCs (real-time qPCR) were measured. HGF and VEGFA protein levels and gene expression showed high inter-patient variability, and no clear pattern emerged. These results can be seen in Supplemental Figures 1 and 2.

**Fig. 6:**
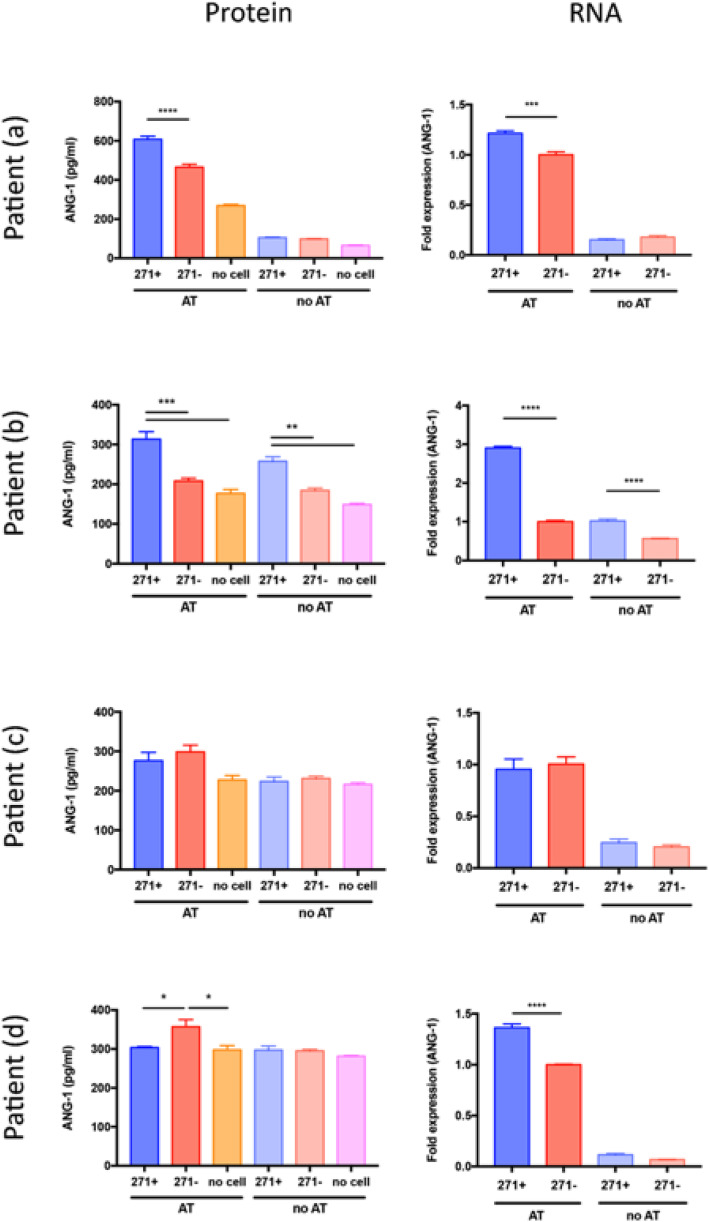
Effect of CD271+ AD-MSCs and AT co-culture on ANGPT1. Both CD271+ and CD271− AD-MSCs were grown with AT, or without AT, for 15 days. Following this, protein levels of ANGPT1 in the co-culture media were measured by ELISA (left column), and RNA levels of ANGPT1 in the AD-MSCs were measured by real-time qPCR (right column). Individual patients are displayed due to high inter-patient variability. *n* = 3 for each patient (experimental replicate)

ANGPT1 protein levels (Fig. 6, left column) showed a consistent pattern within patients (a) and (b). In these patients, ANGPT1 levels were highest in CD271+ cells grown with AT, a significant increase compared to CD271− cells grown with adipose tissue. Patient (a) expressed ANGPT1 at 607.6 ± 16.3 pg/ml in CD271+ AD-MSCs grown with AT, compared to 465.7 ± 14.4 pg/ml in CD271− AD-MSCs grown with AT (*p* < 0.0001), with both of these groups expressing higher levels than in AT alone (268.8 ± 6.1 pg/ml). In patient (b), not only were ANGPT1 levels significantly higher in CD271+ AD-MSCs with AT (313.4 ± 18.9 pg/ml) compared to CD271− AD-MSCs with AT (208.1 ± 7.2 pg/ml, *p* = 0.0002) and AT alone (176.7 ± 10.1 pg/ml, *p* < 0.0001), but CD271+ AD-MSCs grown in the absence of AT also produced higher levels of ANGPT1 than CD271− AD-MSCs grown in the absence of AT (258.3 ± 11.0 pg/ml compared to 184.6 ± 6.0 pg/ml, *p* = 0.0037). In patient (c), there was no significant difference between any group, with ANGPT1 levels between 200 and 300 pg/ml. In patient (d), all groups produced ANGPT1 levels around 300 pg/ml, except for CD271− AD-MSCs grown with AT, which contained ANGPT1 at 357.3 ± 18.3 pg/ml (significant at *p* < 0.05 compared to CD271+ AD-MSCs with AT and AT alone).

ANGPT1 gene expression showed the most consistent pattern between patients for any of the angiogenic genes (Fig. 6, right column). When grown with AT, CD271+ AD-MSCs showed significantly higher ANGPT1 expression compared to CD271− AD-MSCs in patients (a), (b), and (d). This increase was 1.22 ± 0.02 fold in patient (a), 2.90 ± 0.04 fold in patient (b), and 1.36 ± 0.04 fold in patient (d), compared to CD271− AD-MSCs (1 fold in all cases, *p* < 0.001 in all cases). In addition, patient (b) showed a significantly higher expression of ANGPT1 in CD271+ AD-MSCs in the absence of tissue compared to CD271− AD-MSCs (1.02 ± 0.04 fold compared to 0.57 ± 0.01 fold, *p* < 0.0001). Patient (c) displayed no significant differences between cell sorts.

## Discussion

CD271+ cells were confirmed present in human AT by immunocytochemistry and found to colocalise with vascular structures (stained with CD31), confirming previous literature that suggests CD271+ AD-MSCs exist in the adventitial niche of vasculature in human AT [21].

Magnetic sorting enriched the CD271 population from around 20% in unsorted SVF, to over 85% in CD271+ AD-MSCs. This is comparable to other groups who have achieved similar high purities with MACS: Zhang et al. (2015) produced Kupffer cells of purity 95.7% from mouse livers, after a double MACS sort for F4/80+ and CD11c− cells, and Quirici et al. produced a CD271+ population from human AD-MSCs with 88.5% purity by MACS sorting [14, 22]. This is despite prior claims that MACS produces significantly lower purities than FACS [23].

Depletion of CD271 from the negative sort was not as effective, with around 15% expression remaining in CD271− AD-MSCs. There could be a number of reasons for this bleed-through of CD271+ cells into the CD271− population—it is possible that not all CD271+ cells were stained by the microbead antibodies, or that CD271 expression was too low for antibody attachment while being high enough for detection in flow cytometry; rigorous methodological tests would have to be performed to provide an answer. However, for the purposes of this study, it was more pertinent to compare the characteristics and behaviour of the CD271+ population with SVF, rather than an entirely CD271-depleted population. Hence, the CD271− population could be considered practically very similar to the unsorted SVF, with the benefit of having been exposed to the same sorting procedure as the CD271+ population.

The finding that 20% of SVF is composed of CD271+ cells differs from some other literature. Quirici and colleagues found that only 4.4 ± 6.3% of human SVF was composed of CD271+ cells [14], and a team led by Busser found that 11 ± 4% of human SVF was CD271+ [16]. However, a recent study by Beckencamp showed a more similar purity of 19.6% of CD271+ cells in SVF [15]. This range in CD271+ purity is likely due to differences in experimental methodology; most markedly, among these three studies the extraction of SVF from AT was highly variable, with collagenase digestion times varying from 30 to 60 min, and centrifugation speeds varying from 500 to 1200 g. The latter two studies (which found higher purities of CD271) used lower centrifugation speeds, while our study used a much lower centrifugation speed (160*g*). It is likely that as groups further optimise the methodology of SVF extraction, cell survival will improve, making purity evaluations more accurate.

CD271 expression decreased dramatically in the CD271+ population following cell culture. After one passage, the proportion of cells expressing CD271 was reduced from over 80% to below 40%. This is consistent with other studies that show significant changes in AD-MSC phenotype following culture, such as reductions in haematopoietic and inflammatory markers and increases in stromal markers [24–26], and was justification for this study limiting its investigation to passage 0 cells.

CD271+ AD-MSCs were more likely to be CD90+/CD45− compared to both CD271− AD-MSCs and unsorted SVF. As CD90 is a typical marker of a stem cell phenotype, and CD45 is a haematopoietic marker, the fact that CD271+ AD-MSCs were more likely to be CD90+ and CD45− highlights that CD271 is a marker for a more stem cell-like population [27].

RNA sequencing analysis revealed a number of important genetic differences between CD271+ and CD271− AD-MSCs. Notably, principal component analysis revealed that genetic variation between CD271+ and CD271− populations was more significant than between patients. Several hundred genes were significantly altered between the two populations, most pertinently in reactome pathways associated with inflammation and angiogenesis.

Angiogenic genes ANGPT1, ANGPT2, HGF, and VEGFA were expressed at higher levels in CD271+ AD-MSCs, as measured by RNA sequencing. Real-time qPCR confirmed this, with the exception of VEGFA which was not changed significantly. As HGF is a crucial factor in tissue development and angiogenesis [28], and the angiopoietin family are important angiogenic factors in adipose tissue development and remodelling [29, 30], this finding is a good indicator of the proangiogenic potential of the CD271+ population.

The differences between ANGPT1 and ANGPT2 expression are of particular interest. Both these proteins act on the Tie2 receptor, activation of which leads to angiogenesis and anti-inflammation [29]. ANGPT1 is released by mature adipocytes and AD-MSCs, while ANGPT2 is known to be produced by vascular endothelial cells [29]. ANGPT1 activation of the Tie2 receptor leads to an increase in vascular endothelial cell density and can improve wound healing [30], while ANGPT2 has differing effects depending on VEGF presence. In low-VEGF environments, ANGPT2 inhibits the Tie2 receptor and promotes cell death, while in high-VEGF environments, ANGPT2 activates the Tie2 receptor in a unique pathway that produces vascular plasticity and prompts capillary growth [31]. As such, together they are involved in a system that regulates vascular remodelling and angiogenesis in AT (see Supplemental Figure 3).

The fact that RNA sequencing analysis shows significantly higher ANGPT2 expression in CD271+ AD-MSCs suggests that these cells are primed to influence vascular remodelling. Additionally, since ANGPT1 is mainly released by adipocytes, it is not surprising that RNA sequencing of sorted AD-MSCs revealed significant difference in ANGPT2 expression but no significant difference in ANGPT1 expression. Accordingly, later co-culture analysis revealed significantly higher ANGPT1 levels in CD271+ co-culture media, where presumably adipocytes are prompted to release ANGPT1 by CD271+ AD-MSCs.

Although sorted SVF cells have previously been demonstrated to possess proangiogenic properties [32], our findings display proangiogenic potential within an AD-MSC subpopulation sorted without laboratory culture or manipulation. This is important for the potential clinical translation of AD-MSC subpopulation selection.

In addition to the proangiogenic potential we have seen in this CD271+ AD-MSC population, reactome analysis revealed that inflammatory pathways were most significantly changed between CD271+ and CD271− AD-MSCs, suggesting that CD271+ AD-MSCs have anti-inflammatory potential. RNA sequencing revealed that inflammatory genes IL10RA, IL1B, and TNF were significantly downregulated in CD271+ AD-MSCs, which was confirmed by real-time qPCR analysis for IL10RA and IL1B. IL10RA is a key inflammatory receptor, and its overexpression is associated with kidney graft atrophy and fibrosis [33]. IL1B is a key cytokine involved in the inflammatory response to injury in adipose tissue [34], and TNF encourages the activation of M1 macrophages in adipose tissue [35].

Additionally, oncogenic gene WNT2 was expressed several times lower in CD271+ cells compared to CD271− AD-MSCs: inhibitors of WNT2 are considered prime anti-cancer treatments [36]. A reduction in oncogenic factor expression within cellular therapies is particularly desirable for fat grafting treatments following mastectomies, where tissues may be especially susceptible to tumorigenesis.

In the HUVEC tubule formation assay, it was clear from CFSE/RFP co-staining that CD271+ AD-MSCs grew in close proximity with the pseudo-vascular networks. This complements our in vivo findings of CD271+ cells located near vascular structures in AT. Additionally, CD271+ AD-MSCs appeared to produce tubule networks of greater complexity (more tubules and more anastomoses) suggesting that this population would be more therapeutically beneficial in de-vascularised tissues—as smaller, more complex networks arguably indicate more effective microvascular growth, more accurately resembling capillary beds seen in vivo [37].

Growth factors present in Matrigel can potentially influence the results of HUVEC tubule formation assays, and this could have masked changes influenced by CD271+ AD-MSCs. To address this, the HUVEC experiments were also performed with growth factor-depleted Matrigel and the same results were observed.

Analysis of angiogenic protein levels and gene expression was performed in an AD-MSC/AT co-culture model, in order to understand how AD-MSCs and AT react to being in the same environment; both in terms of changes in AD-MSC transcription and the combined protein secretion of both AD-MSCs and AT. The analysis revealed high inter-patient variability, requiring a separation of individual patients to interpret the effects that co-culture had on protein levels and gene expression. Most notably, when CD271+ AD-MSCs were grown with AT, ANGPT1 expression was consistently and significantly higher in these cells compared to CD271− AD-MSCs, in three out of four patients. This higher expression also appeared to prompt higher ANGPT1 protein production by both AT and AD-MSCs, in two of the four patients analysed. In one patient, this increase in ANGPT1 protein was more than cumulative; in other words, AT and AD-MSCs were interacting in a way that prompted more ANGPT1 production than the combination of the amounts they produced on their own. Although the inter-patient variability in this analysis is a cause to question the general applicability of subpopulation selection in surgical techniques, overall these findings support the proangiogenic potential of CD271+ AD-MSCs.

The method of MACS sorting for CD271+ AD-MSCs precludes cell culture or phenotype manipulation and therefore has high potential for clinical translation. Although in vitro cell manipulation can show promising results [32, 38], it is more efficient and safe to avoid laboratory processing and differentiation of cells. MACS sorting of CD34+ cells is already utilised in the surgical treatment of haematological malignancies, so the technology is already translatable [39]. The within-surgery sorting of CD271+ AD-MSCs has great clinical potential as an autologous approach to improve the survival of adipose tissue, especially in large volume fat grafting during breast reconstruction.

## Conclusions

This study demonstrates the proangiogenic potential of CD271+ AD-MSCs and presents this subpopulation as a preferable cellular therapy compared to the more commonly used SVF. Clinical application of cell subpopulation therapies is already established, and the introduction of CD271+ AD-MSCs in therapies such as fat grafting presents a unique and potentially highly translatable method for improving tissue engineering approaches. More work will be required to better understand the patient-to-patient variability in the characteristics of CD271+ AD-MSCs, and extensive in vivo experiments must be performed prior to clinical translation.

## Supplementary Information

**Additional file 1: Supplemental Figure 1.** Effect of CD271+ AD-MSCs and AT co-culture on HGF, Supplemental.

**Additional file 2: Supplemental Figure 2.** Effect of CD271+ AD-MSCs and AT co-culture on VEGFA.

**Additional file 3: Supplemental Figure 3.** The dynamics of angiopoietin in adipose tissue.

**Additional file 4: Supplemental Table 1.** Primers used in real-time qPCR analysis. **Supplemental Table 2.** Greatest gene expression decreases in CD271+ sorted AD-MSCs compared to CD271- sorted AD-MSCs. **Supplemental Table 3.** Greatest gene expression increases in CD271+ sorted AD-MSCs compared to CD271- sorted AD-MSCs. **Supplemental Table 4.** Full reactome pathway analysis results.

## Data Availability

All data generated or analysed during this study are included in this published article [and its supplementary information files]. The data that support the findings of this study are openly available in ArrayExpress at http://www.ebi.ac.uk/arrayexpress/experiments/E-MTAB-8740.

## Abbreviations

AD-MSCs: Adipose tissue-derived mesenchymal stem cells
ANGPT1: Angiopoietin 1
ANGPT2: Angiopoietin 2
AT: Adipose tissue
BM-MSCs: Bone marrow-derived mesenchymal stem cells
CFSE: Carboxyfluorescein succinimidyl ester
ELISA: Enzyme-linked immunosorbent assay
FACS: Fluorescent-activated cell sorting
HGF: Hepatocyte growth factor
HUVECs: Human umbilical vascular endothelial cells
IL10RA: Interleukin 10 receptor A
IL1B: Interleukin 1B
LNGFR: Low affinity nerve growth factor receptor
MACS: Magnetic-activated cell sorting
NGFR: Nerve growth factor receptor
RFP: Red fluorescent protein
SVF: Stromal vascular fraction
TGF-B: Transforming growth factor beta
TNF: Tumour necrosis factor
VEGFA: Vascular endothelial growth factor A
WNT2: Wingless-type MMTV integration site family, member 2
