# Supplementary figures and images for: The angiogenic potential of CD271+ human adipose tissue-derived mesenchymal stem cells
Source: Stem Cell Res Ther. 2021 Mar 2;12:160. doi: 10.1186/s13287-021-02177-0 (PMC7927269; doi:10.1186/s13287-021-02177-0)

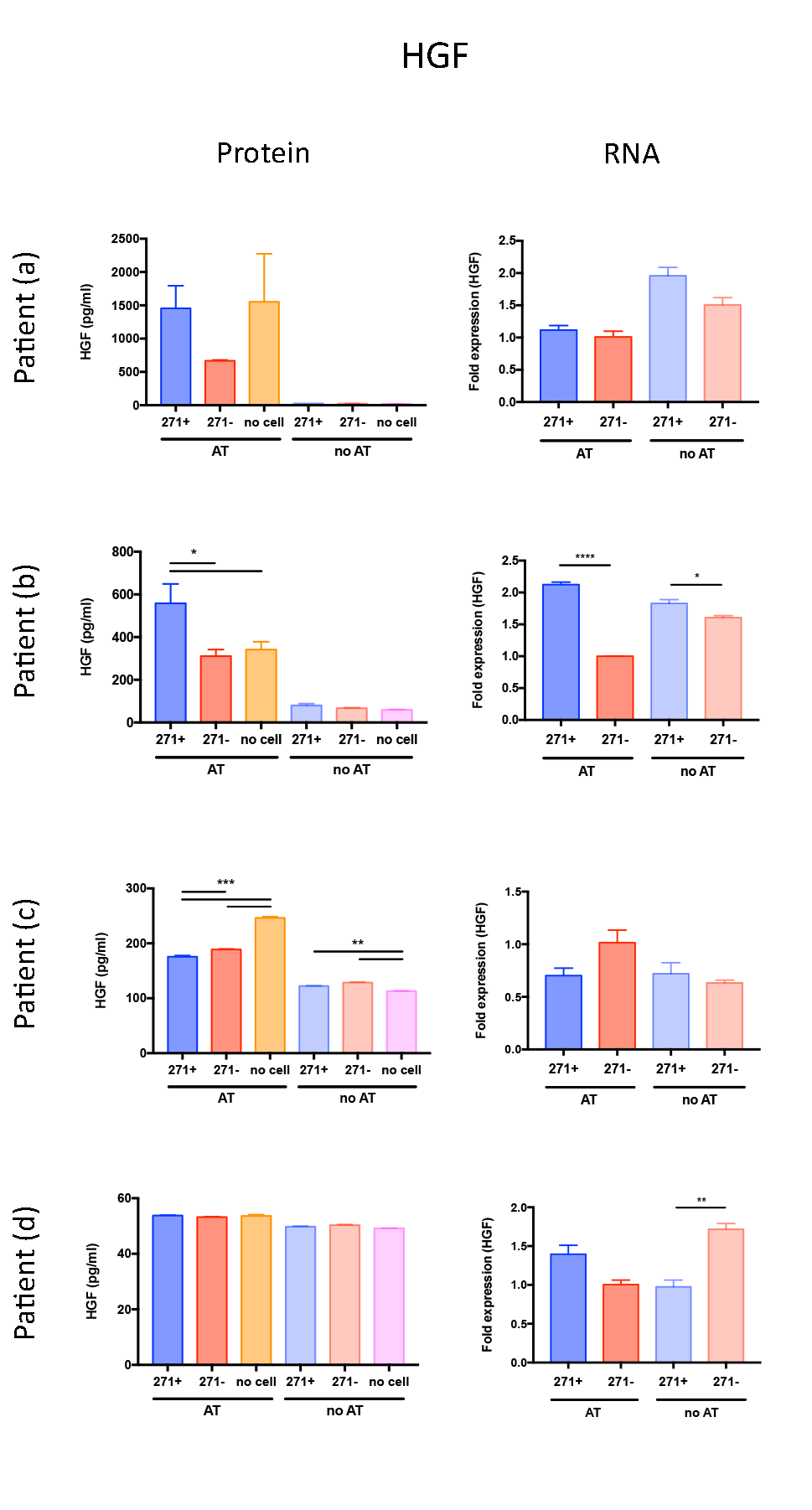

Supplement: Supplementary file 1 — Additional file 1: Supplemental Figure 1. Effect of CD271+ AD-MSCs and AT co-culture on HGF, Supplemental. [file 13287_2021_2177_MOESM1_ESM.tif]

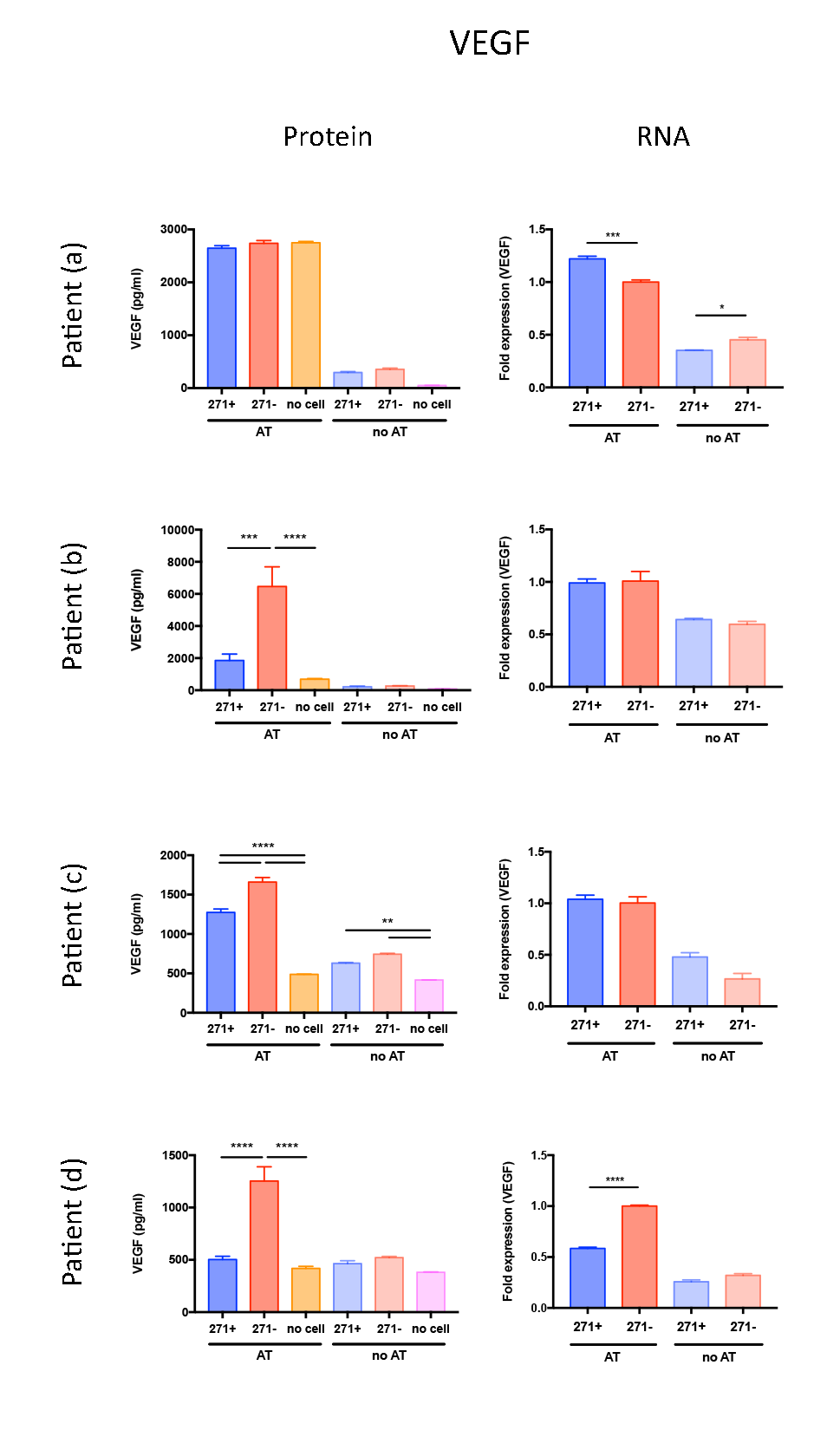

Supplement: Supplementary file 2 — Additional file 2: Supplemental Figure 2. Effect of CD271+ AD-MSCs and AT co-culture on VEGFA. [file 13287_2021_2177_MOESM2_ESM.tif]

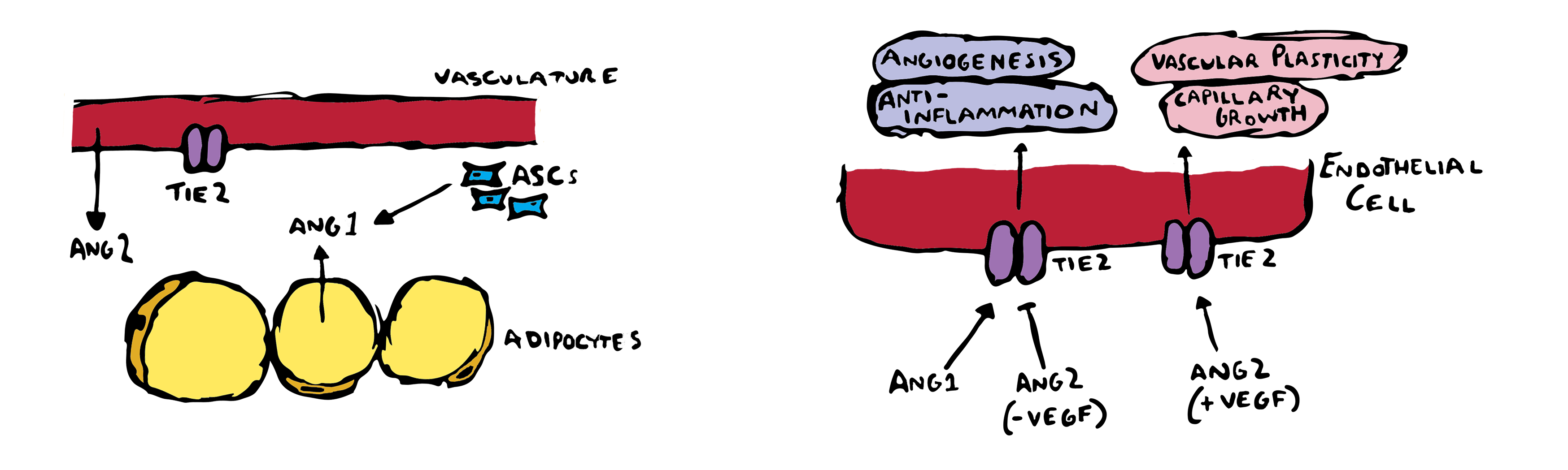

Supplement: Supplementary file 3 — Additional file 3: Supplemental Figure 3. The dynamics of angiopoietin in adipose tissue. [file 13287_2021_2177_MOESM3_ESM.tif]
