# Supplementary material for: The angiogenic potential of CD271+ human adipose tissue-derived mesenchymal stem cells
Source: Stem Cell Res Ther. 2021 Mar 2;12:160. doi: 10.1186/s13287-021-02177-0 (PMC7927269; doi:10.1186/s13287-021-02177-0)
Supplement: Supplementary file 4 — Additional file 4: Supplemental Table 1. Primers used in real-time qPCR analysis. Supplemental Table 2. Greatest gene expression decreases in CD271+ sorted AD-MSCs compared to CD271- sorted AD-MSCs. Supplemental Table 3. Greatest gene expression increases in CD271+ sorted AD-MSCs compared to CD271- sorted AD-MSCs. Supplemental Table 4. Full reactome pathway analysis results. [file 13287_2021_2177_MOESM4_ESM.pdf]

Supplemental Table 1: Primers used in real-time qPCR analysis.

Gene	Sequences	Final concentration (nM)	Product
Adipsin	Fwd: GAAGGTGCAGGTTCTC<br>Rvs: CAGGGCCCAGTGTG	450	Sigma Aldrich (Custom)
Adiponectin	Fwd: GTGAGAAGGGTGAGAA<br>Rvs: CACACTGAATGCTGAG	450	Sigma Aldrich (Custom)
LPL	Fwd: AGGTGATCTTCTGTTCTAG<br>Rvs: GCCGTTCTTTGTTCTG	600	Sigma Aldrich (Custom)
PPARG	Fwd: TCTCCACCTTATTATTCTGA<br>Rvs: CTGTCATAGATAAGCTTCAA	450	Sigma Aldrich (Custom)
VEGF	Fwd: TACCTCCACCATGCCAAGTG<br>Rvs: TGATGATTCTGCCCTCCTCC	450	Sigma Aldrich (Custom)
HGF	Fwd: GATGGCCAGCCGAGGC<br>Rvs: TCAGCGCATGTTTTAATTGCA	450	Sigma Aldrich (Custom)
ANG1	Not disclosed by manufacturer	450	Primer Design (NM_001146)
Oct4	Not disclosed by manufacturer	450	Qiagen (PPH02394E)
Sox2	Not disclosed by manufacturer	450	Qiagen (PPH02471A)
Nanog	Not disclosed by manufacturer	450	Qiagen (PPH17032E)
18s	Not disclosed by manufacturer	240	PrimerDesign (ge-SY-12)

Supplemental Table 2: Greatest gene expression decreases in CD271+ sorted AD-MSCs compared to CD271- sorted AD-MSCs.

Gene	Fold Change	Adjusted p-value	Gene Function
**HBA1**	-178.55	*3.92E-06*	haemoglobin subunit alpha 1
**HBB**	-70.85	*1.40E-112*	haemoglobin subunit beta
**HBA2**	-56.81	*1.08E-19*	haemoglobin subunit alpha 2
**IGKV2D-29**	-39.00	*1.73E-05*	antibody gene
**TMIGD2**	-35.88	*7.07E-05*	transmembrane receptor involved in cell attachment
**TNFRSF13C**	-31.84	*1.25E-05*	TNF family, B-cell receptor
**MS4A2**	-31.65	*1.84E-07*	antibody gene
**IGLV2-14**	-31.10	*3.00E-07*	antibody gene
**IGHV4-61**	-28.45	*2.07E-05*	antibody gene
**NLRP6**	-25.72	*2.98E-05*	involved in inflammatory response
**PRKCQ-AS1**	-25.02	*2.40E-05*	non-coding RNA
**KLB**	-24.70	*2.92E-05*	improves FGF binding
**IGLV2-23**	-22.88	*6.82E-09*	antibody gene
**SCML4**	-22.86	*3.27E-06*	development regulator
**IGLV3-1**	-22.83	*4.23E-06*	antibody gene
**IGHV1-18**	-21.70	*5.56E-18*	antibody gene
**IGHV3-11**	-21.10	*2.61E-10*	antibody gene
**KRT5**	-19.84	*7.53E-06*	keratin gene
**IGLV4-69**	-19.06	*9.46E-05*	antibody gene
**IGHV3-73**	-18.81	*2.78E-09*	antibody gene
**IGKV1-5**	-18.69	*1.86E-08*	antibody gene
**XCR1**	-18.33	*2.12E-06*	chemokine receptor
**IGHV2-5**	-18.03	*9.63E-07*	antibody gene
**PTPRZ1**	-17.89	*2.00E-05*	tyrosine phosphatase receptor, oncogenic in lung
**IGHV3-23**	-17.84	*5.94E-10*	antibody gene
**XCL1**	-17.67	*4.09E-19*	chemokine
**FCRL3**	-17.19	*1.55E-09*	antibody gene
**ASGR2**	-17.00	*2.36E-06*	glycoprotein receptor
**KLRC1**	-16.14	*1.70E-05*	NK cell receptor

Supplemental Table 3: Greatest gene expression increases in CD271+ sorted AD-MSCs compared to CD271- sorted AD-MSCs

Gene	Fold Change	Adjusted p-value	Gene Function
**AC015656.1**	10.27	*2.90E-03*	unknown
**AJ239328.1**	8.55	*2.12E-03*	unknown
**SPRY4-IT1**	7.43	*1.01E-02*	non-coding RNA, upregulated in several cancers, involved in lipid biosynthesis
**GRIK3**	7.03	*5.54E-05*	glutamate receptor
**SLITRK6**	6.85	*7.06E-04*	tyrosine receptor kinase, regulates neurite outgrowth
**CCL19**	5.99	*1.30E-32*	cytokine
**TMEFF2**	5.78	*6.18E-14*	potentially oncogenic transmembrane protein
**AC018926.2**	5.58	*8.33E-03*	non-coding RNA
**TCF23**	5.57	*4.11E-09*	transcription factor
**CLDN1**	5.06	*7.34E-17*	tight junction component
**SCN7A**	4.97	*2.20E-20*	sodium channel subunit
**DKKL1**	4.95	*5.00E-03*	interacts with Wnt signaling pathway
**ABCB11**	4.85	*9.99E-20*	involved in liver function
**IGFN1**	4.80	*2.23E-19*	antibody gene
**NGFR**	4.52	*4.30E-51*	nerve growth factor receptor, or CD271
**RBP5**	4.32	*3.79E-21*	involved in kidney and liver function
**AL645608.1**	4.30	*7.80E-05*	non-coding RNA
**MRAP2**	4.23	*2.90E-07*	involved in body weight regulation
**TUBB3**	4.19	*4.78E-06*	beta tubulin protein, involved in neurogenesis
**PCSK1**	4.14	*3.00E-10*	involved in insulin metabolism
**C7**	4.06	*4.19E-46*	involved in immune response, part of membrane attack complex
**LIPG**	4.06	*2.49E-20*	lipase, involved in lipoprotein metabolism

Supplemental Table 4: Full reactome pathway analysis results.

Pathway	Adjusted P-value	% pathway coverage	-log10 Adjusted P-value
Immunoregulatory interactions between a Lymphoid and a non-Lymphoid cell	2.81792E-28	63.79	27.55
GPCR ligand binding	1.99934E-23	43.53	22.70
Immune System	1.64817E-21	24.55	20.78
Signaling by GPCR	4.0222E-21	32.85	20.40
Class A/1 (Rhodopsin-like receptors)	1.84706E-19	45.45	18.73
GPCR downstream signalling	6.88993E-17	31.74	16.16
Peptide ligand-binding receptors	2.95592E-14	50.48	13.53
Extracellular matrix organization	6.49239E-14	36.78	13.19
Chemokine receptors bind chemokines	5.57581E-13	73.68	12.25
Interleukin-10 signaling	1.80734E-12	68.18	11.74
Hemostasis	2.91094E-12	29.34	11.54
Signal Transduction	7.35286E-12	21.82	11.13
G alpha (i) signalling events	2.01421E-11	33.95	10.70
Phosphorylation of CD3 and TCR zeta chains	2.80414E-09	85.00	8.55
Translocation of ZAP-70 to Immunological synapse	1.34535E-08	88.24	7.87
Integrin cell surface interactions	1.80839E-08	47.44	7.74
Defective B3GALTL causes Peters-plus syndrome (PpS)	2.59087E-08	63.89	7.59
Innate Immune System	3.43802E-08	23.71	7.46
O-glycosylation of TSR domain-containing proteins	5.15768E-08	62.16	7.29
Cytokine Signaling in Immune system	7.43295E-08	25.33	7.13
Platelet activation, signaling and aggregation	1.15664E-07	32.02	6.94
PD-1 signaling	1.39125E-07	76.19	6.86
Cell surface interactions at the vascular wall	1.96959E-07	40.19	6.71
Signaling by Interleukins	2.97264E-07	27.09	6.53
Regulation of Insulin-like Growth Factor (IGF) transport and uptake by Insulin-like Growth Factor Binding Proteins (IGFBPs)	4.79667E-07	41.05	6.32
Generation of second messenger molecules	5.76282E-07	63.33	6.24
Diseases associated with O-glycosylation of proteins	7.7262E-07	49.12	6.11
Binding and Uptake of Ligands by Scavenger Receptors	7.80579E-07	56.41	6.11
O-linked glycosylation	3.35351E-06	40.45	5.47
Interleukin-4 and Interleukin-13 signaling	3.35351E-06	38.78	5.47
Neutrophil degranulation	5.9424E-06	25.52	5.23
ECM proteoglycans	1.17438E-05	43.28	4.93
Non-integrin membrane-ECM interactions	1.51688E-05	45.61	4.82
Adaptive Immune System	2.13711E-05	22.92	4.67
Complement cascade	2.31998E-05	51.28	4.63

Degradation of the extracellular matrix	2.46395E-05	35.34	4.61
G alpha (q) signalling events	3.27599E-05	32.24	4.48
GPVI-mediated activation cascade	5.32506E-05	54.84	4.27
Regulation of Complement cascade	0.000151792	51.52	3.82
Class B/2 (Secretin family receptors)	0.000309832	39.68	3.51
Post-translational protein phosphorylation	0.000406045	36.14	3.39
Initial triggering of complement	0.000432431	64.71	3.36
Collagen chain trimerization	0.000451701	47.37	3.35
Collagen biosynthesis and modifying enzymes	0.000530471	40.00	3.28
RHO GTPases Activate NADPH Oxidases	0.000946019	57.14	3.02
Scavenging by Class A Receptors	0.000946019	61.11	3.02
Interleukin-2 family signaling	0.001012157	45.95	2.99
Syndecan interactions	0.00104475	51.85	2.98
Antimicrobial peptides	0.001224022	52.00	2.91
Neuronal System	0.00129138	25.42	2.89
Constitutive Signaling by Aberrant PI3K in Cancer	0.001330969	38.33	2.88
Platelet degranulation	0.001427354	31.82	2.85
Response to elevated platelet cytosolic Ca2+	0.001620379	31.30	2.79
Other semaphorin interactions	0.001861361	57.89	2.73
Costimulation by the CD28 family	0.00208871	36.36	2.68
Signaling by Receptor Tyrosine Kinases	0.002365889	23.46	2.63
TNFs bind their physiological receptors	0.003986678	50.00	2.40
Common Pathway of Fibrin Clot Formation	0.004445755	66.67	2.35
FCGR activation	0.004503913	66.67	2.35
Collagen formation	0.004780472	33.33	2.32
Molecules associated with elastic fibres	0.00617594	42.86	2.21
cGMP effects	0.00617594	60.00	2.21
Nitric oxide stimulates guanylate cyclase	0.006204883	55.56	2.21
Assembly of collagen fibrils and other multimeric structures	0.009028698	36.36	2.04
Peptide hormone metabolism	0.009318117	35.00	2.03
DAP12 interactions	0.010073357	42.42	2.00
Arachidonic acid metabolism	0.010543674	39.02	1.98
Cross-presentation of particulate exogenous antigens (phagosomes)	0.011598407	75.00	1.94
Netrin-1 signaling	0.013939908	37.78	1.86
Cardiac conduction	0.016014976	29.09	1.80
Elastic fibre formation	0.016463923	38.10	1.78
G alpha (s) signalling events	0.019370156	29.13	1.71
Anchoring fibril formation	0.019370156	57.14	1.71
Potassium Channels	0.023877499	32.81	1.62
FCERI mediated Ca+2 mobilization	0.025873027	40.63	1.59
Metabolism of Angiotensinogen to Angiotensins	0.026404115	53.33	1.58
Platelet Adhesion to exposed collagen	0.031652582	58.33	1.50

Interferon gamma signaling	0.034714647	29.76	1.46
Synthesis of Leukotrienes (LT) and Eoxins (EX)	0.044720257	50.00	1.35
Formation of Fibrin Clot (Clotting Cascade)	0.046546289	43.48	1.33
Dectin-2 family	0.046546289	50.00	1.33
Regulation of TLR by endogenous ligand	0.047998028	53.85	1.32
Activation of C3 and C5	0.047998028	71.43	1.32
Interleukin receptor SHC signaling	0.047998028	43.48	1.32
Laminin interactions	0.047998028	40.00	1.32

Supplemental Figure 1: Effect of CD271+ AD-MSCs and AT co-culture on HGF. Both CD271+ and CD271- AD-MSCs were grown with AT, or without AT, for 15 days. Following this, protein levels of HGF in the co-culture media were measured by ELISA (**left column**), and RNA levels of HGF in the AD-MSCs was measured by real-time qPCR (**right column**). Individual patients are displayed due to high inter-patient variability. n=3 for each patient (experimental replicate).

Supplemental Figure 2: Effect of CD271+ AD-MSCs and AT co-culture on VEGFA. Both CD271+ and CD271- AD-MSCs were grown with AT, or without AT, for 15 days. Following this, protein levels of VEGFA in the co-culture media were measured by ELISA (**left column**), and RNA levels of VEGFA in the AD-MSCs was measured by real-time qPCR (**right column**). Individual patients are displayed due to high inter-patient variability. n=3 for each patient (experimental replicate).

Supplemental Figure 3: The dynamics of angiopoietin in adipose tissue. Angiopoietin 1 is released by mature adipocytes and AD-MSCs, while angiopoietin 2 is known to be produced by vascular endothelial cells. Both proteins act on the Tie2 receptor; activation of which leads to angiogenesis and anti-inflammation. Angiopoietin 1 activation of the Tie2 receptor leads to an increase in vascular endothelial cell density and can improve wound healing, while angiopoietin 2 has differing effects

depending on VEGF presence. In low-VEGF environments, angiopoietin 2 inhibits the Tie2 receptor and promotes cell death; while in high-VEGF environments, angiopoietin 2 activates the Tie2 receptor in a unique pathway that produces vascular plasticity and prompts capillary growth. As such, together they are involved in a relatively complex system that regulates vascular remodelling and angiogenesis in AT.
